# P-1005. Real-World Effectiveness of Fidaxomicin in Preventing Recurrence of Initial Community-Associated Clostridioides difficile Infection

**DOI:** 10.1093/ofid/ofaf695.1202

**Published:** 2026-01-11

**Authors:** christopher J Myers, Adam J Hawco, Runda Dahhan, Christine Hurley, Edwin van Wijngaarden, Ghinwa Dumyati

**Affiliations:** University of Rochester, Rochester, NY; NY Emerging Infections Program, URMC, West Seneca, New York; University of Rochester, Rochester, NY; University of Rochester, Center for Community Health and Prevention, Rochester, New York; University of Rochester, Rochester, NY; New York Emerging Infections Program and University of Rochester Medical Center, Rochester, New York

## Abstract

**Background:**

In 2021, ISDA guidelines recommended fidaxomicin over vancomycin as first line treatment of Clostridioides difficile infection (CDI) based on clinical trials showing reduced recurrence. However, real-world effectiveness studies of fidaxomicin primarily in hospitalized and high-risk populations showed mixed results. Evidence of fidaxomicin effectiveness for community associated (CA) CDI in outpatient settings remains limited. The study aim is to evaluate the real-world effectiveness of fidaxomicin for initial CA-CDI.

**Methods:**

We used data from population-based surveillance of CDI in Monroe County, NY (2020-2024), as part of the CDC Emerging Infections Program. CA CDI was defined as a first episode with no healthcare exposure in the prior 12 weeks. Adults (≥ 18 years) who completed an uninterrupted course of oral therapy were included. Clinical data, inpatient treatment and recurrence at 8 weeks and 90 days were abstracted from medical records. Recurrence rates were compared between fidaxomicin and vancomycin treated groups using bivariate and multivariable log-binomial regression models.

**Results:**

Among 1193 cases with initial CA-CDI,146 (12.2%) received fidaxomicin and 1047 (87.8%) vancomycin. Groups were similar in sex, comorbidities and prior PPI/H2 blocker use. Cases receiving fidaxomicin were more likely to be aged 40-59 (32% vs. 23.3%). In hospital treatment was comparable (20.5% vs. 18.8%). Recurrence at 8 weeks and 90 days was 8.2% and 8.9% for fidaxomicin, and 14.9% and 17.8% for vancomycin, respectively (p < 0.05). Crude models showed that vancomycin was associated with a higher risk of 8-week recurrence (RR: 1.81; 95% CI: 1.03–3.18) and 90-day recurrence (RR: 2.00; 95% CI: 1.17–3.41). After adjustment, the risk remained elevated for 8-week recurrence (RR: 1.72; 95% CI: 0.98–3.03) and 90-day recurrence (RR: 1.89; 95% CI: 1.10 – 3.23).

**Conclusion:**

Fidaxomicin was associated with a lower recurrence rate than vancomycin in CA-CDI with a statistically significant benefit at 90 days. These findings support the current IDSA guidelines and highlight the real-world advantage of fidaxomicin for initial CA-CDI management.

**Disclosures:**

All Authors: No reported disclosures

